# Normalization of ChIP-seq data with control

**DOI:** 10.1186/1471-2105-13-199

**Published:** 2012-08-10

**Authors:** Kun Liang, Sündüz Keleş

**Affiliations:** 1 Department of Statistics and Actuarial Science, University of Waterloo, Waterloo, Ontario N2L 3G1, Canada; 2 Department of Statistics, University of Wisconsin-Madison, Madison, WI 53706, USA; 3 Department of Biostatistics and Medical Informatics, University of Wisconsin-Madison, Madison, WI 53706, USA

## Abstract

**Background:**

ChIP-seq has become an important tool for identifying genome-wide protein-DNA interactions, including transcription factor binding and histone modifications. In ChIP-seq experiments, ChIP samples are usually coupled with their matching control samples. Proper normalization between the ChIP and control samples is an essential aspect of ChIP-seq data analysis.

**Results:**

We have developed a novel method for estimating the normalization factor between the ChIP and the control samples. Our method, named as NCIS (Normalization of ChIP-seq) can accommodate both low and high sequencing depth datasets. We compare statistical properties of NCIS against existing methods in a set of diverse simulation settings, where NCIS enjoys the best estimation precision. In addition, we illustrate the impact of the normalization factor in FDR control and show that NCIS leads to more power among methods that control FDR at nominal levels.

**Conclusion:**

Our results indicate that the proper normalization between the ChIP and control samples is an important step in ChIP-seq analysis in terms of power and error rate control. Our proposed method shows excellent statistical properties and is useful in the full range of ChIP-seq applications, especially with deeply sequenced data.

## Background

Genome-wide protein-DNA interactions including transcription factor binding and epigenomic modifications play a crucial role in the programming of cell specific gene expression. Therefore, their genome-wide mapping with the ChIP-seq (Chromatin immunoprecipitation followed by sequencing) technology can significantly advance our ability to understand biology and human diseases. As a result, ChIP-seq is now routinely used in many applications, e.g., [1-3].

In a ChIP-seq experiment, the DNA fragments from binding sites of a target protein or from sites of specific histone modifications are enriched through immunoprecipitation. These sites can be sharp point sources in transcription factor binding, or long and diffused regions in some histone modifications, or combination of both in RNA polymerase-DNA interactions [4]. Then sequenced ends (reads) of millions DNA fragments are aligned to a reference genome to identify enrichment sites with over-abundance of reads. In ChIP samples, there are large number of fragments generated from non-specific “background” regions throughout the genome. Thus, the reads in a ChIP sample can be considered as a mixture of enrichment signal reads and background noise reads [5].

Early studies without the use of control samples have assumed uniform background read distribution when assessing the significance of enrichment sites [6,7]. However, regions with high read counts do not necessarily contain enrichment sites. Many follow-up studies have shown that the distribution of reads is far from uniform and is affected by many factors, including GC content [8,9], mappability [10], chromatin structure and copy number variation [11], among others. The most effective approach to account for these known and other unknown biases is to include a matching control sample that is generated either from input DNA or by using non-specific antibody.

The ChIP and control samples usually are sequenced at different depths (total number of reads). A common strategy for making the samples “comparable” is to linearly scale according to the sequencing depth ratio. Because of the mixture nature of ChIP sample, it is reasonable to align/normalize only the background reads of the ChIP sample with respect to the control sample. Hence, an appropriate normalization involves the estimation of the background reads proportion (*Π*_0_) among ChIP sample reads and the corresponding ChIP/control normalization factor. The proper estimation of the normalization factor is important for finding weak enrichment sites, especially for those sites whose enrichment ratio is between the sequencing depth ratio and the true normalization factor. The existence of weak enrichment sites has been experimentally validated and shown to be biologically meaningful [5].

The normalization factor is a critical parameter of most ChIP-seq data analysis programs that can utilize control samples. For example, CisGenome [12] and PeakSeq [10] explicitly use the normalization factor to estimate *p*-values under Binomial distribution. MACS [13], SPP [14], and USeq [15], among many others, use the normalization factor to linearly scale the control sample for comparison with the ChIP sample. Furthermore, many programs (MACS, SPP, SISSRs [16] and others) estimate false discovery rate (FDR) using a sample-swapping method as follows. After computing an enrichment statistic for each non-overlapping region, the FDR can be estimated as *R*_*I *_(*s*)/*R*_*C *_(*s*), where *R*_*I *_(*s*) and *R*_*C *_(*s*) are the numbers of enriched regions called on the control sample and the ChIP sample, respectively, using the same threshold *s* on the enrichment statistics. To make the statistics in ChIP and control samples comparable, a normalization factor is implicitly used in the FDR estimation. Therefore, the normalization factor is a crucial parameter of enrichment site detection and error rate control in ChIP-seq data analysis.

Last but not least, the estimation of background reads proportion *Π*_0_ is of scientific interest itself. *Π*_0_ can be viewed as an overall quality indicator which is related to the specificity of the antibody used in an experiment, experimental design, and other experimental protocols. We have observed that *Π*_0_ can vary from 0.3 to close to 1 in many ChIP-seq datasets. Unless the number of truly enriched regions is small, *Π*_0_ close to 1 indicates the scarcity of enrichment reads and the need for better antibody or protocol, or both.

Many ChIP-seq data analysis programs (CisGenome, SPP, PeakSeq and CCAT [5]) have proposed methods for estimating the normalization factor; however, their performances under diverse set of settings have not been studied. Most of the above methods are intuitively appealing; but many rely on ad-hoc tuning parameters.

In this paper, we develop a novel normalization method and compare it with existing methods through data-driven simulations. We further demonstrate that our method leads to better estimation accuracy, FDR control, and power than other methods.

## Methods

Empirical studies show that the background (non-signal) parts of the ChIP sample and the control sample exhibit a (approximately) linear relationship [10]. We further demonstrate such linear relationship in human, worm *C.elegans* and yeast *S.cerevisiae* ChIP-seq datasets in [Additional file 1: Section 1]. We begin our exposition by reviewing existing normalization strategies that are well documented in the published journal articles and then present our method.

### Existing methods for estimating ChIP to control normalization factor

Suppose there are *N*_1 _and *N*_2 _uniquely aligned reads for the ChIP and the control samples, respectively. According to the signal-noise model proposed by [5], the reads in the ChIP sample can be decomposed into *Π*_0_*N*_1_ background and (1 −* Π *_0_)*N*_1 _enriched signal reads. Then the correct ChIP/control ratio should be $r=\frac{\Pi_{0}N_{1}}{N_{2}}$. We will refer to *r* as the normalization factor in the rest of the paper.

To estimate the normalization factor, the commonly used set-up is to divide reference genome into non-overlapping bins of width *w*, numbered from 1 to *m*. Let *n*_1*i *_and *n*_2*i *_denote the total number of reads in the *i*th bin in the ChIP sample and the control sample, respectively, and *n*_*i *_=* n*_1*i*_ + *n*_2*i *_denote the total number of ChIP and control reads for bin *i*. If the knowledge of which bins are within background regions were given to us by an oracle, then a natural estimator of the ChIP/control ratio would be

(1) $$\hat{r}=\frac{\underset{i\in B}{\sum }n_{1i}}{\underset{i\in B}{\sum }n_{2i}},$$

where *B* represents the index set of the background bins provided by the oracle. Each existing normalization factor estimation method employs a different approach for estimating *B*. Given that enrichment sites tend to have high read counts, bins with small total counts are more likely to belong to background. CisGenome sets bin-width *w* = 100 bp and uses the bins with low total counts as background. Specifically, *B*_*w *_(*t*) ={*i*:*n*_*i *_≤* t*} and the total threshold *t* is set to 1. As implied by this definition, *B*_*w *_(*t*) depends on the choice of *w* and *t*. Another idea, similar in spirit but operating on the opposite direction, is to exclude bins with high read counts. SPP estimates the background regions by excluding highly “enriched” regions with a small *p*-value either in the ChIP sample or the control sample under uniformity assumption on the reads. Specifically, SPP sets *w* = 1 Kbp and *B *= {*i*:min(*p*_1*i*_,*p*_2*i*_) >* c*}, where *p*_1*i*_and *p*_2*i *_are the Poisson *p*-values for testing whether the *i*th ChIP and control bin read counts are generated from an uniform background read distribution, and the threshold *c* is set to 10^−5^.

CCAT estimates *B* and the normalization factor in an iterative fashion where *B* is estimated based on reads from the positive strand and *r* is updated using reads from the negative strand through (1). More specifically, in the *j*th iteration, $B=\{i:n_{1i+}<{\hat{r}}^{\left(j\right)}n_{2i+}\}$, where ${\hat{r}}^{\left(j\right)}$ is the current estimate of the normalization factor which is initialized at the sequencing depth ratio and *n*_1*i* + _ and *n*_2*i* + _ are ChIP and control positive strand read counts in bin *i* with bin-width *w* = 1 Kbp. The algorithm iterates till convergence.

A related method, PeakSeq, first defines enriched regions by using a certain threshold of FDR on the height of the ChIP sample read profile. Instead of using (1), PeakSeq then excludes a proportion (*P*_*f*_) of bins that overlap with putative enrichment sites defined in the first step and utilizes the slope of linear regression of ChIP against control bin counts (with *w* = 10 Kbp) as the normalization factor.

All the above methods attempt to approximate the background region in some intuitive way; however they rely on tuning parameters which are set in an ad-hoc fashion. The suggested bin-width *w* ranges from 100 bp to 10 Kbp. Utilized definitions of the background regions *B* depend on arbitrary thresholds on an array of parameters, e.g., total count, *p*-value, and FDR. The same procedure with different tuning parameters may lead to drastically different estimates. In an application of PeakSeq [10], the estimates of the normalization factor changes from 1.24 to 0.96 when the exclusion proportion *P*_*f *_changes from 0 to 1. Furthermore, there aren’t any established guidelines for optimally setting the tuning parameters.

Two other methods (MOSAiCS by [9] and ZINBA by [17]) have adopted similar mixture-regression approaches by modeling ChIP counts in background and signal regions as functions of multiple covariates (including but not limited to, control read count, mappability and GC content). However, their regression coefficients of the control read count are unlikely to be suitable for estimating normalization factor. This is because their regression functions are usually more complicated than a simple linear relationship between ChIP and control read counts. In MOSAiCS, the linear regression is on some power of the control read count; whereas in ZINBA, there can be interaction terms involving control read count.

### Estimating normalization factor: NCIS

We propose a new method named as NCIS (Normalization of ChIP-seq). Our method extends CisGenome’s estimator by choosing the optimal value of bin-width *w* and the threshold of total read counts *t* in a data-adaptive manner. In general, the smaller the total count threshold *t*, the more likely that bins with small total counts, i.e., *n*_*i *_≤* t*, are from background regions, and thus, the normalization factor estimated from (1) by treating these bins as background tends to have smaller bias (deviation of the normalization factor estimate to the true value). CisGenome sets *t* to 1, the smallest possible non-zero total count, so that bias can be minimized. On the other hand, using larger *t* will increase the size of *B*_*w *_(*t*) and reduce variance (spread of the normalization factor estimates). Statistically, the choice of *t* represents the trade-off between bias and variance.

**Figure 1:**
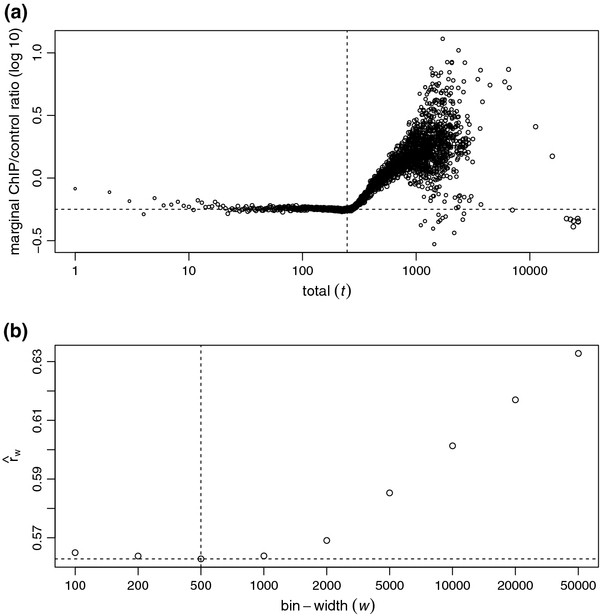
**ChIP/control ratio as a function of total count for *C.elegans *data.** (a) Marginal ChIP/control ratio against total count, both in log (10) scale, from a *C.elegans* ChIP-seq dataset of transcription factor PHA-4 [18]. Sizes of the plotting circles are proportional to log (10) of numbers of reads. Vertical dash line marks the total count selected by NCIS to estimate the normalization constant. Horizontal dash line marks the normalization factor estimate from NCIS. (b) Normalization constant as a function of bin-width. Vertical dash line marks the bin-width selected by NCIS to estimate the normalization constant. Horizontal dash line marks the normalization factor estimate from NCIS.

We now motivate our method through a real ChIP-seq study. Figure 1a shows the marginal ChIP/control ratio ($r_{m}(t)=\underset{i:n_{i}=t}{\sum }n_{1i}/\underset{i:n_{i}=t}{\sum }n_{2i}$) against the total count (*t*) with *w* = 500 bp for a *C.elegans* ChIP-seq dataset of transcription factor PHA-4 [18]. On the left half of the figure where *t* is small, the ratio estimates fall around a horizontal line, and the variability increases as *t* becomes small. This observation illustrates that the reads from the bins with small total counts are mostly from background regions and their marginal ChIP/control ratios are similar. On the right half of Figure 1a, there is a strong ascent of marginal ratios which indicates the significant infusion of enrichment signal reads into the ChIP reads.

Our method takes into account the above observations and operates as follows. First, all reads will be shifted towards their 3’ end by *l*/2, where *l* is the average DNA fragment length available either through experimental protocol or computational estimation (e.g., [13,14,16]). Because it is sufficient to use only nonempty bins, we filter out bins with zero total count (*n*_*i *_= 0). For any fixed bin-width *w*, define ${\hat{r}}_{w}(t)=\underset{i\in B_{w}(t)}{\sum }n_{1i}/\underset{i\in B_{w}(t)}{\sum }n_{2i}$ as in (1) and *B*_*w *_(*t*) ={*i*:*n*_*i *_≤* t*} as in the previous subsection. We search for a total threshold *t* instead fixing it at a pre-specified constant. In most ChIP experiments, it is reasonable to believe that the vast majority of the genomic landscape are background regions. Therefore, to avoid large variation in estimating ${\hat{r}}_{w}(t)$ when *t* and size of _*Bw*_(*t*) are small, we start searching for *t* at the upper quartile of the non-zero total counts. Specifically, our estimate of the normalization factor with a fixed bin-width *w* is ${\hat{r}}_{w}={\hat{r}}_{w}(t_{w}^{*})$ where $t_{w}^{*}=\min\{t:{\hat{r}}_{w}(t)\geq {\hat{r}}_{w}(t-1),|B_{w}(t)|\geq 0.75m_{w}\}$ and _*mw*_ is the total number of bins. That is, ${\hat{r}}_{w}$ is the first ${\hat{r}}_{w}(t)$ estimate that is larger than or equal to its previous one and is based on more than three quarters of the bins. For example, the vertical dash line in Figure 1a marks the $t_{w}^{*}$ selected by NCIS; it separates the background regions on the left from the signal regions on the right.

It is reasonable to set the other tuning parameter, bin-width *w*, close to the width of the enrichment site so that there are clear contrasts between the read counts of the ChIP and the control samples when the bins coincide with enrichment sites. However, without knowledge of the exact locations of enrichment sites and also in the settings where the lengths of enrichment sites vary, it is not possible to put the bin boundaries tightly around enrichment sites. As a result, enriched sites are likely to be split into two or more bins, and it is advantageous to use small bin-width to gain resolution if the sequencing depth is high enough. Hence, we search over a grid of bin-width {*w*_1_,*w*_2_,…,*w*_*n*_} such that *w*_1_<* w*_2_ < … <* w*_*n*_ and stop at the first bin-width that satisfies ${\hat{r}}_{w_{i+1}}\geq {\hat{r}}_{w_{i}}$ and use ${\hat{r}}_{w_{i}}$ as our final estimate $\hat{r}$. That is, $\hat{r}={\hat{r}}_{w_{i^{*}}}$ where $i^{*}=\min\{i:{\hat{r}}_{w_{i+1}}\geq {\hat{r}}_{w_{i}}\}$. Note that the values of ${\hat{r}}_{w_{i}}$ are bound to increase because the normalization factor equals to sequencing depth ratio (the upper limit of normalization factor) when *w* equals to the total genome size. As an example, the normalization constant estimates (${\hat{r}}_{w}$) of the *C.elegans* data are plotted in Figure 1b as a function of bin-width (*w*), and the vertical dash line indicates the bin-width that reaches the minimum of the ${\hat{r}}_{w}$ values. The default values for bin-width grid values are set at {100, 200, 500, 1k, 2k, 5k, 10k} to cover the range of bin-width used by existing methods.

## Results and discussion

### A comparison of statistical properties of normalization factor estimators

In this section, we used the yeast ChIP-seq study of [19] to generate data in our data-driven simulations. A parallel simulation study that is based on a *C.elegans* dataset is presented in the [Additional file 1: Section 3]. We selected the yeast dataset because it is one of the deepest sequenced publicly available ChIP-seq datasets in terms of genome coverage. The yeast genome is about 250 times smaller than that of the human such that high coverage can be easily achieved on yeast with relatively small number of reads. The control sample of segregant 1 (SEG1) has more than 4.2M uniquely aligned reads and is one of the deepest sequenced control samples in the study. With an average fragment length of 200 bp, 4.2M fragments amount to about 70X coverage on the yeast genome. As a comparison, 20M reads (common output of number of uniquely aligned reads from one lane on an Illumina Hi-seq sequencer) for a human sample is equal to about 1X coverage. We randomly split the SEG1 control sample into two halves and subsampled 1/*d* of each, where *d* is a subsampling divisor parameter. One of the subsamples was treated as control, and the other half was mixed in with simulated reads from *p* enriched sites and treated as a ChIP sample. Using a high coverage yeast dataset and a subsampling strategy, we can investigate the performance of methods under a spectrum of coverage. For example, the coverage achieved by 20M reads on a human sample is roughly equal to the coverage on a simulated yeast control sample with *d* = 30. As the cost of sequencing decreases rapidly, we can look into the “future” of ChIP-seq on large mammalian genomes by studying the performances of methods at small values of *d*.

We simulated reads for enriched regions in three different scenarios. Setting 1 mimics ChIP-seq data of transcription factors where enrichment reads concentrate in sharp peaks. Our set-up is similar to the simulation setting of [12]. More specifically, reads for enrichment sites were simulated from *N*(*μ*_*i*_*σ*^2^) with *μ*_*i*_ randomly assigned along the genome and *σ*^2 ^= 900. The number of reads of each site followed an exponential distribution with mean *c*·*N*_2_/*p*so that, on average, we spiked-in *c* times the total number of control sample reads (*N*_2_). Parameter *c*, which represents the proportion of signal reads relative to the background was set to 0.2, 0.5 and 1 to represent weak to strong overall binding signal strength. The value of *p* was set to 1000, based on the results of [19] which identified about 1000 binding sites for the transcription factor Ste12 in various strains of yeast. The subsampling divisor, *d*, took values in {1,2,5,10,20,50,100} to represent different depths of coverage. The simulation was repeated 100 times for each combination of *c* and *d*.

**Figure 2:**
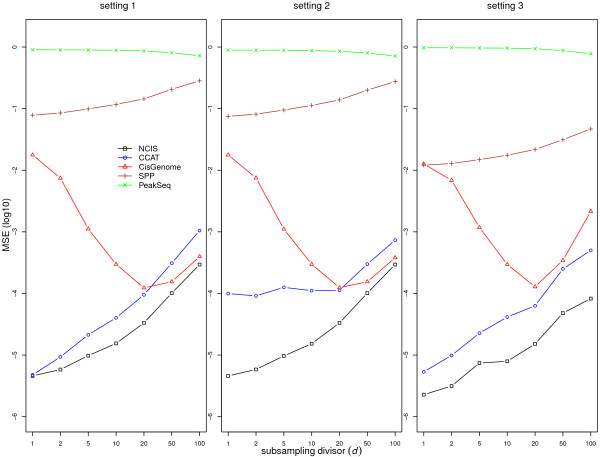
**Statistical properties of normalization factor estimators.** Mean and MSE (log10) for estimating the normalization factor in simulation setting 1 (left), setting 2 (middle) and setting 3 (right) with *c* = 1. The true value of the normalization factor is 1.

In this simulation study, we compare our estimator (NCIS) with estimators proposed in CisGenome, SPP, CCAT, and PeakSeq. The exclusion proportion parameter *Pf * in PeakSeq was set at 0 to simplify its computation. Left panel of Figure 2 displays the log (10) of mean squared error (MSE) for setting 1 (transcription factor binding) with *c* = 1. We chose MSE as our comparison metric because it considers both the bias and the variance of the estimators compared to true normalization factor. Overall, our NCIS estimator has the smallest MSE among all the methods. PeakSeq estimator is the worst in estimation precision, followed by SPP. CisGenome estimator has second best MSE when sequencing depth is low; however its performance deteriorates when sequencing depth is high. The performances of all the estimators except PeakSeq and CisGenome improve with the increase of sequencing depth. The rest of the results (*c* = 0.2 and 0.5) for setting 1 are similar and are provided in [Additional file 1: Figure S6 and S7].

In setting 2, we study the impact of artifacts on normalization constant estimation. High-throughput sequencing experiments, including ChIP-seq experiments, are complex biochemical and computational processes, and it is common that the sequencing data contain various artifacts. Some examples of artifact regions where the control sample has significantly higher read count than the ChIP sample are displayed and discussed in [Additional file 1: Section 1]. We first simulated reads as in setting 1, then we generated artifacts according the patterns observed in the above examples. More specifically, we randomly chose 20 locations along the genome and generated artifact reads on these locations in the control sample such that the total number of artifact reads is a small percentage (0.5%) of the original control sample sequencing depth. Figure 2 (middle panel) illustrates that most methods are not affected significantly by the presence of artifacts. However, the performance of CCAT is much worse than in setting 1, indicating its lack of robustness with respect to these artifacts.

In setting 3, we simulated the enrichment reads to resemble histone modifications and polymerase binding where enrichment reads are spread out on large regions. We allocated enriched reads uniformly on *p* = 50 regions. The length of each region was generated uniformly between 5–15 Kbp. The distribution of the number of reads of each region, signal/background proportion *c* and subsampling divisor *d* were the same as in above transcription factor set-up. Right panel of Figure 2 displays the results for *c* = 1 while the results for *c* = 0.2 and 0.5 are provided in [Additional file 1: Figure S6 and S7]. Our method remains the best in terms of MSE.

We also simulated various levels of the true normalization factor (e.g., 0.3, 0.5, 0.8, 1.2, 2 and 3) by using different subsampling divisors on split halves. The results are similar to those presented here for the true normalization factor of 1 and hence are not reported. In term of estimation precision, the order of the methods (from best to worst) is: NCIS, CCAT, CisGenome, SPP, and PeakSeq.

### FDR control and power

We evaluate the impact of using different normalization factor estimators on FDR control and power with simulated data similar to the previous setting 2 with the same split-subsampling procedure and the addition of artifacts. However, we generated the binding site locations and signal strength differently. We first called peaks using SPP at FDR level 0.05 and obtained 1572 putative binding locations from the SEG1 yeast data. Then the signal strength at each site was estimated as its ChIP count minus its corresponding (normalized) control count. At each iteration, we randomly sample 1000 sites from the total 1572 and let the number of reads at each site follows a Poisson distribution with mean equal to the estimated signal strength. Because the binding site locations and signal strengths are obtained from a real ChIP data, a more realistic power result can be achieved.

FDR control is achieved through the sample-swapping method as discussed in the Background Section. To obtain putative binding site locations (peak-calling), we employed a simple two-stage search strategy. In the first stage, we partitioned the yeast genome into 100 bp non-overlapping bins and retained only bins with Binomial *p*-value smaller than or equal to some liberal threshold, for example, 0.05. Then in the second stage, we merged nearby retained bins into putative regions and searched each region to locate the 20 bp bin with the highest ChIP bin count and used the center of this bin as our prediction of the putative binding site. For each binding site, we extended 110 bp from the site location to both directions and formed a binding region. Then we used the ChIP and control read counts in the binding region to compute a Binomial *p*-value as the enrichment statistic for the binding site. More specifically, for each normalization factor estimator, the Binomial probability *p* when comparing the ChIP sample to the control sample is computed as $\hat{r}/(1+\hat{r})$, where $\hat{r}$ is their respective estimate of normalization factor. Further details for computing Binomial *p*-value can be found in [10,12]. This peak-calling procedure was first performed with the ChIP sample versus the control sample to obtain a list of putative binding sites and their statistics, and repeated one more time with the control sample versus the ChIP sample to obtain a list of control binding sites and their statistics. Then a number of putative binding sites were declared as true binding sites such that the empirical FDR (#control sites/#ChIP sites) did not exceed certain nominal level (0.05) using the same *p*-value threshold. A site was classified as false positive if the predicted location was 100 bp away from its closest true binding site. Note that there were few declared binding sites located between 50 bp to 100 bp away from true binding sites, so any choice between 50 bp and 100 bp would yield similar results.

Simulations for FDR estimation of the sample-swapping method have been performed in [5], and our simulation differs from theirs in two major ways. First, simulations in [5] only evaluated the FDR estimation over a range of nominal FDR levels with a fixed sequencing depth, while our simulations evaluate FDR control over varying sequencing depths. Second, FDRs computed in [5] are on the basis of 1 Kbp non-overlapping regions, while we classified predicted binding sites by their distance to their closest true binding sites. Our false positive criteria is more accurate and relevant because a single 1 Kbp region can hold multiple binding sites and one or more these sites can be close to the boundary of two adjacent regions such that both regions would be regarded as true binding regions.

**Figure 3:**
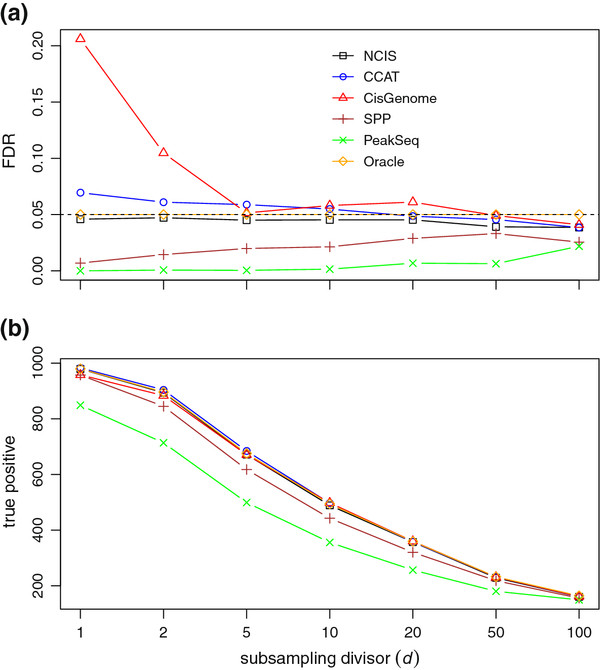
**FDR control and power.** FDR control with the sample-swapping method. (a) compares FDR levels with different normalization factor estimators. (b) Power comparison between between FDR control at 0.05 level with different normalization factor estimators.

As a comparison, we also performed peak calling when the normalization factor is set to its true value of 1 and refer to this method as the Oracle. FDR is controlled at the target level of 0.05, and Figure 3a displays the means of the realized FDR for various methods. The FDR values of the Oracle are close to the nominal value of 0.05 (the median of the differences is 0.002 while the median of the standard errors is 0.0012). For display purpose, the Oracle FDR values are plotted at the expected value of 0.05, and other methods are adjusted accordingly. CisGenome and CCAT fail to control FDR at various sequencing depths, especially when the sequencing depth is high. CisGenome’s FDR values can be drastically larger than nominal level at high sequencing depths because its normalization estimate becomes unreliable and highly variable. CCAT’s failure to control FDR is due to the negative bias resulting from the artifacts. Among all the methods, the FDR values of NCIS are the closest to the Oracle. Figure 3b shows the power (number of true positive) of all methods against different subsampling divisors/sequencing depths. Among all methods that can control FDR at the nominal level (NCIS, SPP and PeakSeq), NCIS is the most powerful method and is indistinguishable from the Oracle. On average, NCIS is about 6% more powerful than the second best (SPP) across different sequencing depths.

### Application

#### Yeast Ste12 data

We applied our method on the ChIP-seq data of yeast strain SEG1 in [19] and estimated *Π*_0_, the background proportion in ChIP sample, to be 0.763. The original analysis was performed by MACS, which assumes the sequencing depth ratio as the normalization factor. To make results comparable with the original analysis, we modified the latest stable version of MACS (1.4.1) such that it can utilize user specified normalization factors through an additional input parameter. The estimated normalization factor (*r*), background proportion (*Π*_0_) and number of detected binding sites under two different criterion are listed in Table 1. The first criteria “#peaks by *p*-value” refers to the number of peaks detected by MACS with its default *p*-value threshold of 1*e*-5, and the second criteria “#peaks by FDR” refers to the number of peaks detected by MACS with FDR controlled at 0.05 level. The other parameters of MACS were set to be the same as in the original analysis. Using different normalization factors has a dramatic impact on the power to detect binding sites and the estimation of FDR. This is because it is difficult to call peaks in ChIP sample but relatively easy to do so in control sample with a conservative normalization constant such as the sequencing depth ratio. For example, MACS only declared 1 significant binding region at FDR level 0.05 in contrast to 1322 significant binding regions with NCIS estimate at the same FDR threshold. On the other hand, MACS estimated the FDR for the most significant 1000 peaks to be 0.23, while the FDR for the top 1000 peaks was estimated as 0.04 using the NCIS estimate. The roughly 6 fold difference in the estimated FDR was caused by a relative small change in the normalization factor estimates. This example illustrates the importance of the normalization factor estimator. A recent paper [3] also pointed out that MACS overestimated FDR 7.5 fold in their study, and that it is highly likely that the incorrect normalization factor is the major contributing factor. CisGenome’s estimate of normalization factor is very conservative. This is because the SEG1 strain was deeply sequenced and as a result, there are only 240 bins whose ChIP and control read count total equal to 1. Hence, CisGenome’s estimate is expected to be highly variable due to the small sample size to estimate normalization factor when sequencing depth is high. This is consistent with our observation in Figure 1 and the simulation results.

**Table 1:** Comparison of normalization factor estimators on yeast strain SEG1 through the MACS algorithm

	$\hat{r}$	${\hat{\Pi }}_{0}$	**#peaks by***p***-value**	**#peaks by FDR**	
NCIS	1.265	0.763	1844	1322	
CCAT	1.173	0.707	1943	1723	
SPP	1.370	0.826	1736	688	
CisGenome	1.553	0.937	1547	4	
PeakSeq	1.674	1.009	1431	1	
MACS	1.658	1	1449	1	

The fourth column contains the numbers of detected peaks with *p*-value ***≤ ***1* e*-5. The fifth column contains the numbers of detected peaks at FDR level 0.05.

**Figure 4:**
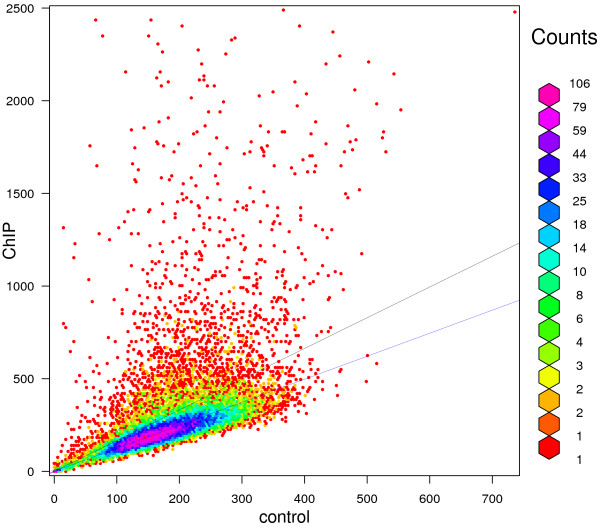
**ChIP vs control bin counts for yeast strain SEG1.** ChIP versus control bin counts for yeast strain SEG1 plotted with bin-width of 500 bp. The upper black line represents the sequencing depth ratio, and the lower blue line the NCIS normalization factor estimate.

To further illustrate the differences between different normalization factors, we plotted the ChIP versus the control bin counts with bin-width *w* = 500 bp in Figure 4. In this plot, different colors indicate different densities of bins which are annotated at the right-hand side. There are many bins with relatively high ChIP counts due to the enrichment signal. The slope of the upper black line is the sequencing depth ratio, and majority of bins (83.7%) appear below this line. We should expect less than 50% of bins to appear below the normalization factor line because binding regions have smaller than 0.5 probability to exhibit a ChIP count/control count ratio below the normalization factor. The NCIS normalization factor is represented by the lower blue line, which passes right through the densest area of bins and has 49.7% of bins below the line.

#### Human NF*κ*B data

We next compared different normalization estimators on a human NF*κ*B ChIP-seq dataset in [20], where the genome-wide binding of transcription factor NF*κ*B was extensively studied on multiple cell lines. As one of the deepest sequenced cell lines among the data collected, cell line GM12878 has 48.5 and 24.8 million uniquely mapped reads in the ChIP and the control samples, respectively. Table 2 shows estimates of normalization factor from different methods.

**Table 2:** **Comparison of normalization factor estimators on NF *κ *B ChIP-seq data of cell line GM12878**

	**NCIS**	**CCAT**	**SPP**	**CisGenome**	**PeakSeq**
$\hat{r}$	1.758	1.657	1.834	2.123	1.883
${\hat{\Pi }}_{0}$	0.895	0.844	0.933	1.082	0.958

The sequencing depth ratio is 1.963.

**Figure 5:**
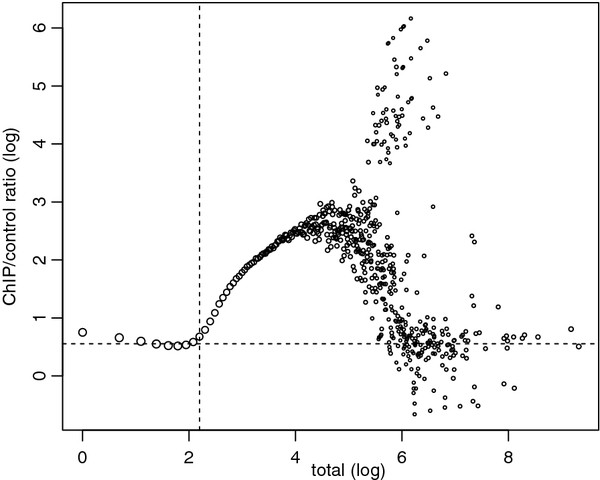
**ChIP/control ratio as a function of total count for human NF***κ***B data.** NF*κ*B marginal ChIP/control ratio against total with bin-width of 100 bp, both in natural log scale. Sizes of the plotting symbols are proportional to the log (10) of the number of reads. Horizontal dash line indicates the NCIS estimate of the normalization factor. Vertical dash line represents the NCIS total count threshold (*t*_*w*_^∗^).

Figure 5 displays the marginal ChIP/control ratio against total read counts. We observe that the NF*κ*B data is noisier compared to the yeast data and exhibits violations of the signal-noise model assumption. That is, some bins have larger control reads than expected as illustrated on the right bottom corner of the plot. This phenomenon can arise due to various artifacts in the ChIP-seq experiments, for example, PCR over-amplification in control sample. Indeed, we traced most outliers to a 5 Kbp region in chromosome 8. The read count per nucleotide is displayed in [Additional file 1: Figure S5]. This plot indicates that these are artifacts which are over-amplified in the control sample. The CCAT estimator is susceptible to such artifacts and can have downward bias in estimating the normalization factor. On the other hand, NCIS and CisGenome only utilize bins with low total counts and are robust to such artifacts. SPP is also robust to these artifacts to some degree due to its filtering of bins with large ChIP and control read counts. In this dataset, CisGenome’s estimate of normalization factor is larger than the sequencing depth which is an unreasonable outcome for the normalization factor.

### Software

R package (http://www.r-project.org) for NCIS is available in [Additional file 2].

### Discussion

As the sequencing technology improves rapidly over time, deeply sequenced data sets will become more common. We demonstrated in our simulation and application studies that CisGenome estimator’s performance deteriorates when sequencing depth increases. In one unpublished and deeply sequenced *E.coli* dataset (Courtesy of Professor Tricia Kiley, UW Madison), we observed that CisGenome estimator was not applicable because every mappable bin had more than one read. Although we studied the FDR in a balanced (*r* = 1) simulation setting, our analytical results also support FDR control for unbalanced data in the [Additional file 1: Section 4].

## Conclusions

In this study, we systematically evaluated the available ChIP-seq normalization factor estimators through data-based simulations. All existing estimators rely on some ad-hoc tuning parameters, which may be crucial to the final estimate. Our NCIS method is data-adaptive and has better estimation precision (smaller MSE) than existing methods over a wide range of sequencing depths, and for both sharp and diffused ChIP signals. Given the importance of normalization factor in evaluating protein-DNA binding efficiency and the power of detecting protein-DNA binding sites while achieving proper error control, we expect our method to contribute significantly to the ChIP-seq research and applications.

## Competing interests

The authors declare that they have no competing interests.

## Authors’ contributions

KL designed the study, wrote the NCIS package, conducted statistical analyses, and drafted the manuscript. SK designed the study and drafted the manuscript. Both authors read and approved the final manuscript.

## Supplementary Material

Additional file 1 **Section 1.** Illustration of linearity between ChIP and control samples. Section 2. Simulation results for the normalization factor estimators at different settings with yeast data. Section 3. Simulation results for the normalization factor estimators with *C.elegans* data. Section 4. FDR control for unbalanced data, [21]. Click here for file

Additional file 2 NCIS R Package. Click here for file
