# Supplementary material for: Normalization of ChIP-seq data with control
Source: BMC Bioinformatics. 2012 Aug 10;13:199. doi: 10.1186/1471-2105-13-199 (PMC3475056; doi:10.1186/1471-2105-13-199)
Supplement: Additional file 1 — Section 1. Illustration of linearity between ChIP and control samples. Section 2. Simulation results for the normalization factor estimators at different settings with yeast data. Section 3. Simulation results for the normalization factor estimators with C.elegans data. Section 4. FDR control for unbalanced data, [21]. [file 1471-2105-13-199-S1.pdf]

Supplement Materials for

Normalization of ChIP-seq data with control

Kun Liang and Sündüz Keleş

1 Linearity between ChIP and control samples

We demonstrate that a proper control sample correlates linearly with the background parts of its corresponding ChIP sample. In the following examples, we first draw the original ChIP vs control bins counts to show the over-abundance of high ChIP count bins due to binding signals. Then we filter the strong binding signals by calling peaks with SPP (Kharchenko et al., 2008) at FDR 0.1 level and exclude all the bins that intersect with the peaks. Then the remaining ChIP and control bin counts show clear linear trend and are roughly symmetric around the normalization factor estimated through NCIS.

1.1 Yeast data

Zheng et al. (2010) studied transcription factor Ste12 in various yeast strains. The ChIP and control samples bin counts of one of the strains (segregant 1) are plotted in Figure 1a. After peak-calling and exclusion of the bins that intersect with peaks, the remaining ChIP and control bin counts are plotted in Figure 1b.

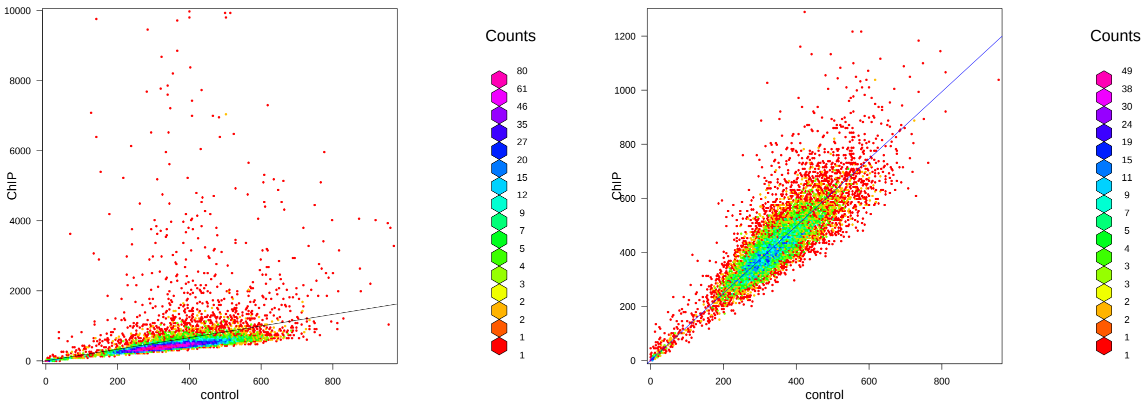

(a) original (b) filtered

Figure 1: Linearity between ChIP and control samples in yeast data. The black line in (a) represents sequencing depth ratio. The blue line in (b) represents the NCIS normalization factor estimate. The plots are based on bin width of 1 Kbp.

1.2 *C. elegans* data

Zhong et al. (2010) studied binding of the transcription factor PHA-4 in *C.elegans* at first stage of larval development under starvation. The ChIP and control samples

bin counts are plotted in Figure 2a. After peak-calling and exclusion of the bins that intersect with peaks, the remaining ChIP and control bin counts are plotted in Figure 2b.

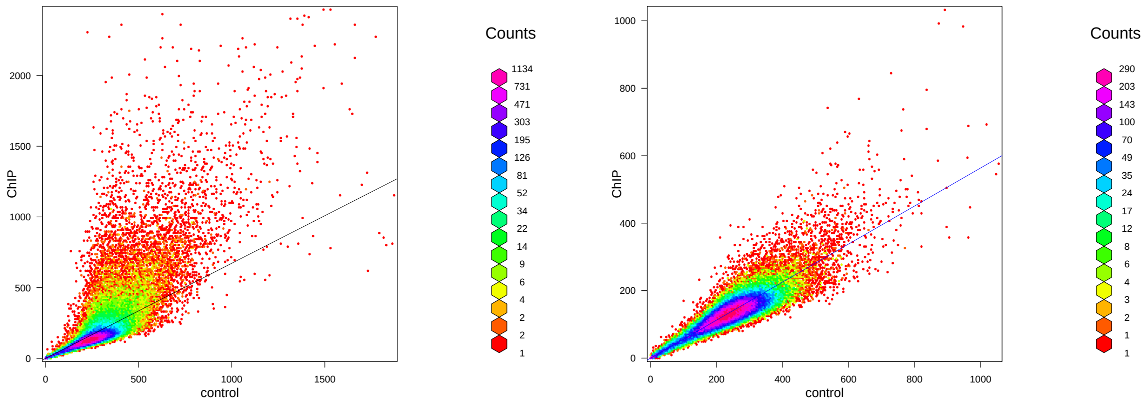

(a) original (b) filtered

Figure 2: Linearity between ChIP and control samples in *C. elegans* data. The black line in (a) represents sequencing depth ratio. The blue line in (b) represents the NCIS normalization factor estimate. The plots are based on bin width of 1k bp.

1.3 Human NFκB data

Kasowski et al. (2010) studied binding of the transcription factor NFκB in ten human cell lines. The ChIP and control samples bin counts of cell line GM12878 are plotted in Figure 2a. After peak-calling and exclusion of the bins that intersect with peaks, the remaining ChIP and control bin counts are plotted in Figure 2b.
There are some noticeable outliers where control sample has much larger read counts than ChIP sample. For example, the rightmost point in Figure 3b can be traced back to a region on chromosome 1, where the read count per nucleotide is plotted in Figure 4. The reads on each strand are not concentrated on a single nucleotide but rather on a stretch of 30 nucleotides. The footprint of a typical transcription factor binding site on a single strand should be larger than the average fragment length, which is about 200 bp in this case. Also given the fact that the control sample has much larger read count than the ChIP sample, these reads are most likely the result of some artifacts. Similarly, most outliers that appeared below the normalization line in Figure 5 of the main text can be traced to a region in chromosome 8 and is plotted in Figure 5.

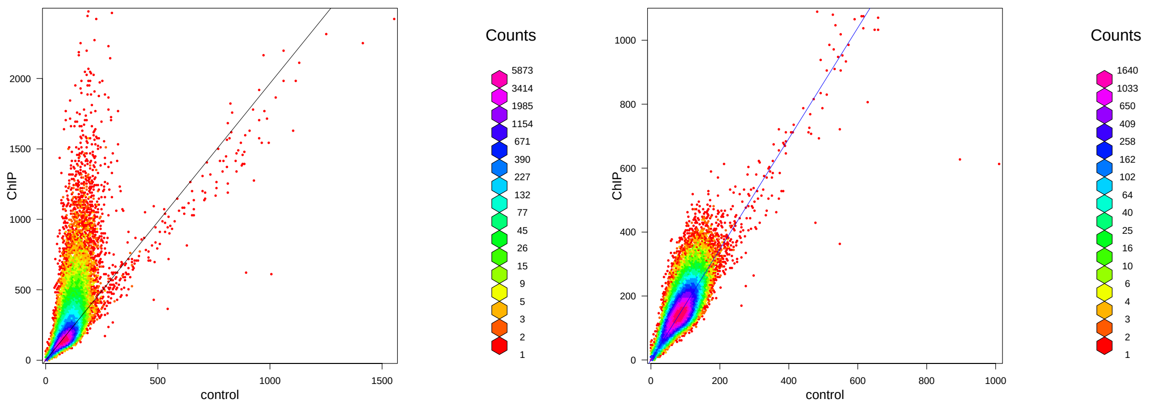

(a) original (b) filtered

Figure 3: Linearity between ChIP and control samples in Human NFκB data. The black line in (a) represents sequencing depth ratio. The blue line in (b) represents the NCIS normalization factor estimate. The plots are based on bin width of 10k bp.

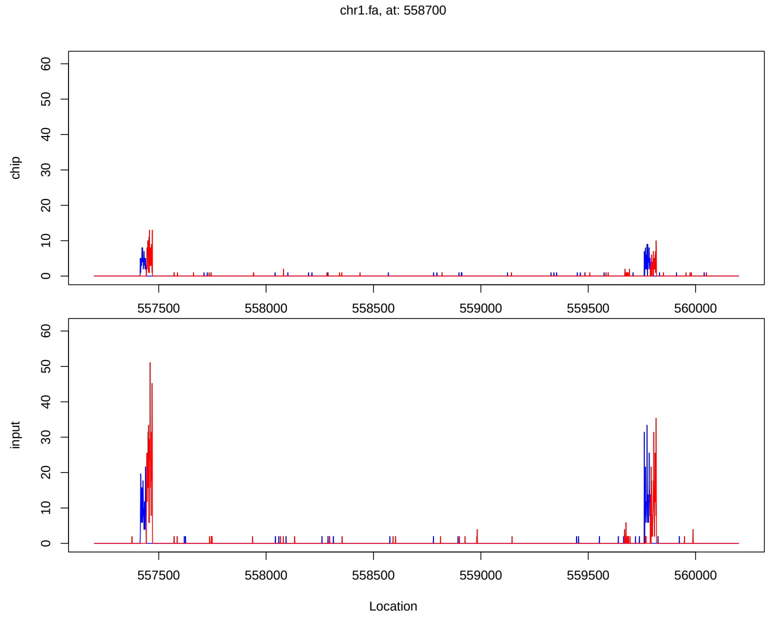

Figure 4: Read count per nucleotide for chromosome 1, [557200, 560200]. The blue line indicates positive strand reads and the red line indicates negative strand. The read counts between ChIP and control samples are normalized by their sequencing depths.

2 Simulation Results for the Normalization Factor with Yeast Data

3 Simulation Results *C.elegans* Data

3.1 Precision

In this section, we present simulation results based on a deeply sequenced *C.elegans* ChIP-seq dataset (Zhong et al., 2010). In this dataset, the control sample of the

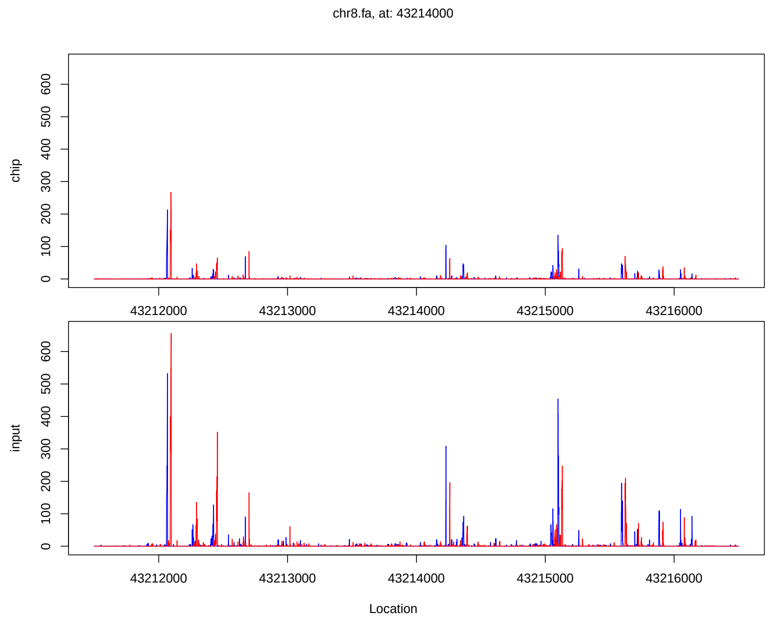

Figure 5: Read count per nucleotide for chromosome 8, [43211500, 43216500]. The blue line indicates positive strand reads and the red line indicates negative strand. The read counts between ChIP and control samples are normalized by their sequencing depths.

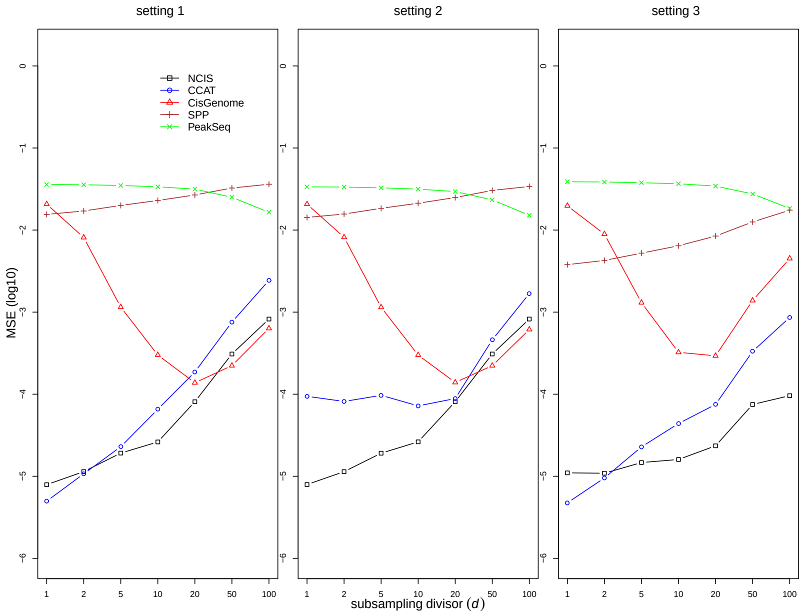

Figure 6: MSE (log10) for estimating the normalization factor in yeast data of Section 2 in simulation setting 1-3 with $c = 0.2$.

first stage of larval development under starvation has about 26.8 million uniquely mapped reads. The *C.elegans* genome has roughly 100 million base pairs and is about 33 times smaller than the human genome. Thus, in terms of genome coverage, the *C.elegans* dataset is on the same order as the yeast dataset used in the main text. We performed simulations with parameters identical to those used in the main text

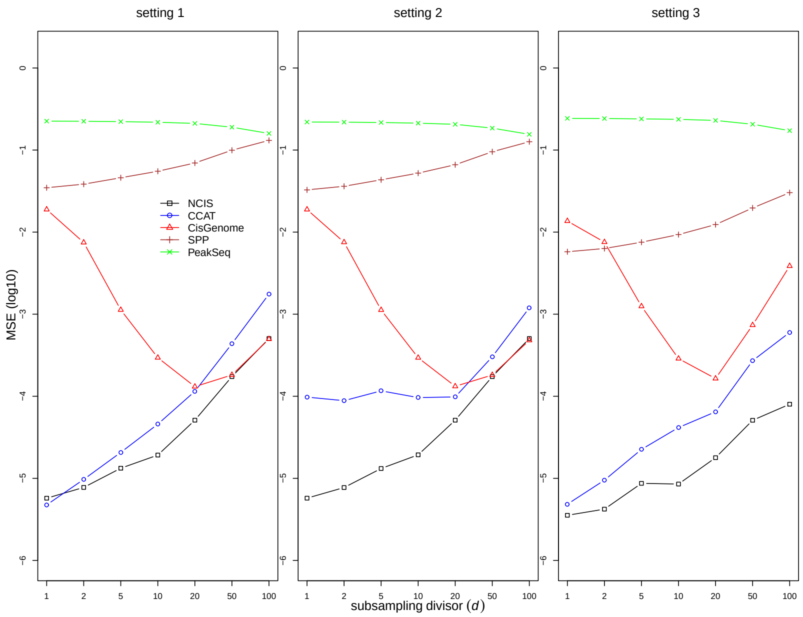

Figure 7: MSE (log10) for estimating the normalization factor in yeast data of Section 2 in simulation setting 1-3 with $c = 0.5$.

for the yeast data. Although the absolute values of MSE change due to the changes in genome, the relative performances of different normalization factor estimators remain mostly the same.

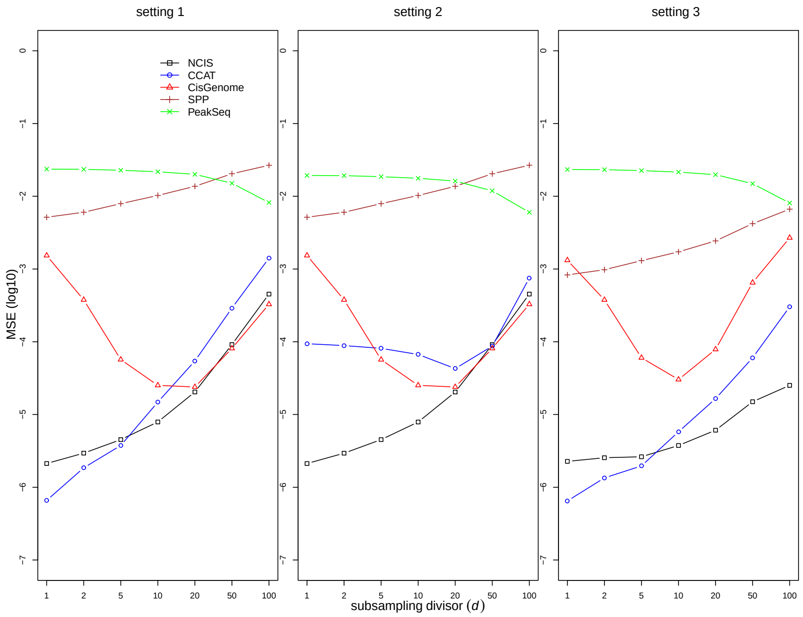

Figure 8: MSE (log10) for estimating the normalization factor in *C.elegans* data of Section 3 in simulation setting 1-3 with $c = 0.2$.

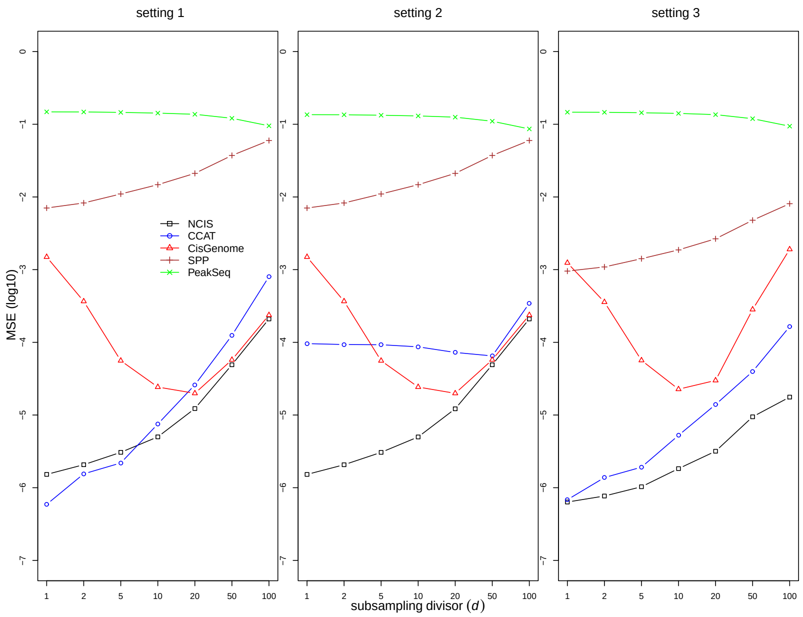

Figure 9: MSE (log10) for estimating the normalization factor in *C.elegans* data of Section 3 in simulation setting 1-3 with $c = 0.5$.

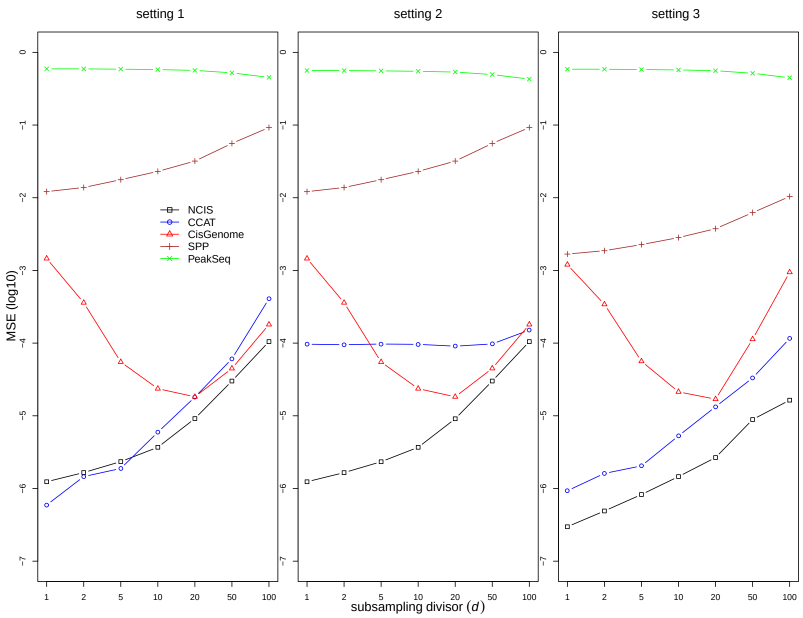

Figure 10: MSE (log10) for estimating the normalization factor in *C.elegans* data of Section 3 in simulation setting 1-3 with $c = 1$.

3.2 FDR control and power

We follow the same procedure as in Section "FDR control and power" in the main text except we selected 5000 sites from candidate sites of 7725 predicted from the *C.elegans* data (SPP FDR level 0.01) in each iteration. The added artifacts leads to a negative bias in PeakSeq normalization estimation due to its regression approach's

sensitivity to the influential points. As a result, FDR with PeakSeq is out of control. On average, NCIS is about 14% more powerful than SPP, which is the only other method maintain proper FDR control, across different sequencing depths.

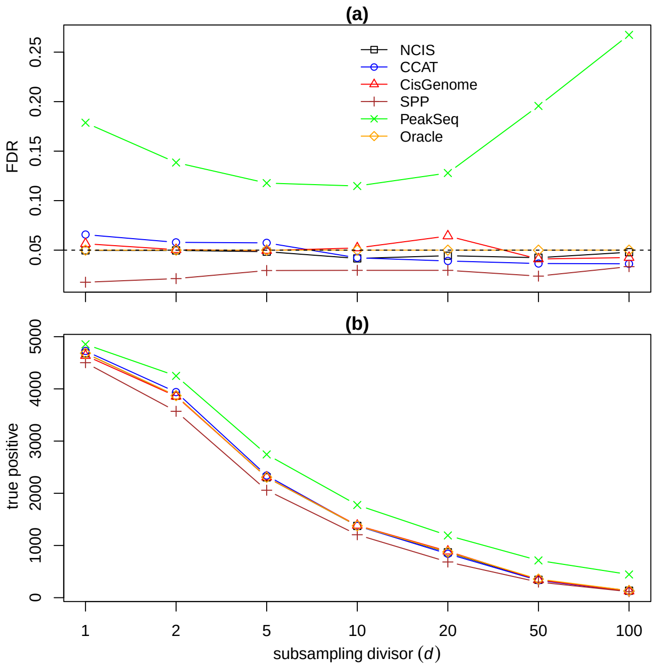

Figure 11: MSE (log10) for estimating the normalization factor in *C.elegans* data in simulation setting 1-3 with $c = 1$.

4 FDR control for unbalanced data

The purpose of normalization in ChIP-seq analysis is to make the ChIP and the control samples comparable. We define the ChIP and the control samples as balanced when $\pi_0 N_1 = N_2$, or equivalently, $r = 1$. The balance can be judged in practice by checking whether $\hat{\pi}_0 N_1 = N_2$ after obtaining $\hat{\pi}_0$, an estimate of π_0. The theory of the sample-swapping method for estimating FDR in ChIP-seq data analysis was studied by Xu et al. (2010) under a balanced setting. However, almost all ChIP-seq datasets exhibit imbalance. One strategy to deal with unbalanced data is to subsample the larger of the ChIP and control samples to achieve balance. This strategy has been advocated and practiced in Xu et al. (2010) and Smagulova et al. (2011). The obvious drawback of the subsampling strategy is that part of the samples will not be utilized. To address this issue, a strategy has been proposed in Xu et al. (2010) to resample multiple copies of balanced data and merge the results so that all reads of the samples can have a chance to contribute to the final result. We hypothesize that by incorporating the normalization factor into significance score, the loss of data in the the subsampling strategy and the added computational complexity of resampling strategy can be avoided. Let $g(a_i, b_i, r)$ be a normalized significance score function based on ChIP count a_i, corresponding control count b_i of region i and normalization factor r when comparing the ChIP sample to the control sample. Define E as the collection of nucleotides at which ChIP signal is enriched. We now focus on the positive FDR (pFDR) which is proposed in Storey (2003) and can be defined in ChIP-seq context as

$$\mathrm{pFDR}(s) = \Pr(i \subset \overline{E} | g(a_i, b_i, r) \geq s),$$

where s is a significance threshold.

Theorem 1 Under the conditions:
(a) $\Pr(g(a_i, b_i, r) \geq s | i \subset \overline{E}) \approx \Pr(g(b_i, a_i, 1/r) \geq s | i \subset \overline{E})$ for large s and
(b) $\Pr(g(a_i, b_i, r) \geq s | i \not\subset \overline{E}) \gg \Pr(g(b_i, a_i, 1/r) \geq s | i \not\subset \overline{E})$,
the estimated pFDR at a threshold s can be approximated as

$$\mathrm{pFDR}(s) = \frac{\#\{g(b_i, a_i, 1/r) \geq s\}}{\#\{g(a_i, b_i, r) \geq s\}}$$

for large s.

The first condition requires the normalized significance scores to have similar tail distributions in background regions of the ChIP and control samples. The second condition assumes good separation of the significance scores of $g(a_i, b_i, r)$ and $g(b_i, a_i, 1/r)$ when the region i is not entirely within background regions. When $r = 1$, the approximation in condition (a) is exact.

Proof of Theorem 1

$$\begin{aligned}\mathrm{pFDR}(s) &= \mathrm{P}(i \subset \overline{E} | g(a_i, b_i, r) \geq s) \\ &= \frac{\mathrm{P}(g(a_i, b_i, r) \geq s | i \subset \overline{E}) \mathrm{P}(i \subset \overline{E})}{\mathrm{P}(g(a_i, b_i, r) \geq s)}\end{aligned}$$

$$\approx \frac{P(g(b_i, a_i, 1/r) \geq s | i \subset \overline{E}) P(i \subset \overline{E})}{P(g(a_i, b_i, r) \geq s)},$$

where the last step is by condition (a). From condition (b), we also have

$$\frac{P(g(b_i,a_i,1/r) \geq s | i \not\subset \overline{E}) P(i \not\subset \overline{E})}{P(g(a_i,b_i,r) \geq s)} \ll \frac{P(g(a_i,b_i,r) \geq s | i \not\subset \overline{E}) P(i \not\subset \overline{E})}{P(g(a_i,b_i,r) \geq s)} \leq 1$$

Thus,

$$\begin{aligned} \text{pFDR}(s) &\approx \frac{P(g(b_i, a_i, 1/r) \geq s | i \subset \overline{E}) P(i \subset \overline{E})}{P(g(a_i, b_i, r) \geq s)} \\ &\quad + \frac{P(g(b_i, a_i, 1/r) \geq s | i \not\subset \overline{E}) P(i \not\subset \overline{E})}{P(g(a_i, b_i, r) \geq s)} \\ &= \frac{P(g(b_i, a_i, 1/r) \geq s)}{P(g(a_i, b_i, r) \geq s)}, \end{aligned}$$

which can be approximated as

$$\frac{\#\{g(b_i, a_i, 1/r) \geq s\}}{\#\{g(a_i, b_i, r) \geq s\}}$$

References

Kasowski, M., Grubert, F., Heffelfinger, C., Hariharan, M., Asabere, A., Waszak, S., Habegger, L., Rozowsky, J., Shi, M., Urban, A., et al. (2010), "Variation in transcription factor binding among humans," *Science*, 328, 232–235.

Kharchenko, P., Tolstorukov, M., and Park, P. (2008), "Design and analysis of ChIP-seq experiments for DNA-binding proteins," *Nature biotechnology*, 26, 1351–1359.

Smagulova, F., Gregoretti, I., Brick, K., Khil, P., Camerini-Otero, R., and Petukhova, G. (2011), "Genome-wide analysis reveals novel molecular features of mouse recombination hotspots," *Nature*, 472, 375–378.

Storey, J. (2003), "The positive false discovery rate: A Bayesian interpretation and the q-value," *Annals of Statistics*, 31, 2013–2035.

Xu, H., Handoko, L., Wei, X., Ye, C., Sheng, J., Wei, C., Lin, F., and Sung, W. (2010), "A signal–noise model for significance analysis of ChIP-seq with negative control," *Bioinformatics*, 26, 1199–1204.

Zheng, W., Zhao, H., Mancera, E., Steinmetz, L., and Snyder, M. (2010), "Genetic analysis of variation in transcription factor binding in yeast," *Nature*, 464, 1187–1191.

Zhong, M., Niu, W., Lu, Z., Sarov, M., Murray, J., Janette, J., Raha, D., Sheaffer, K., Lam, H., Preston, E., et al. (2010), "Genome-wide identification of binding sites defines distinct functions for Caenorhabditis elegans PHA-4/FOXA in development and environmental response," *PLoS Genet*, 6, e1000848.
